# Connatal Urinary Ascites in a Female Preterm

**DOI:** 10.1155/2017/6760218

**Published:** 2017-10-11

**Authors:** Barbara Brunner, Elisabeth Ralser, Elisabeth D'Costa, Kathrin Maurer, Ursula Kiechl-Kohlendorfer, Elke Griesmaier

**Affiliations:** ^1^Department of Paediatrics II, Medical University of Innsbruck, Innsbruck, Austria; ^2^Department of Gynaecology and Obstetrics, Medical University of Innsbruck, Innsbruck, Austria; ^3^Department of Radiology, Medical University of Innsbruck, Innsbruck, Austria

## Abstract

**Background:**

Connatal urinary ascites is rare in females without associated malformations and occurs following bladder rupture.

**Case Presentation:**

A female very preterm was delivered by caesarean section because of abnormal Doppler findings. The mother suffered from viral pneumonia requiring intensive care in the third trimester of pregnancy. Serial fetal ultrasound examinations showed a megacystis and ascites. Postnatally, pronounced isolated ascites was drained and its urinary nature was confirmed. The bladder leak was demonstrated when blue dye, instilled via a Foley catheter, appeared in the ascitic drain. After removal of the catheter spontaneous micturition was unremarkable. A micturating cystourethrogram showed spontaneous closure of the bladder leak.

**Conclusion:**

The female infant experienced fetal bladder rupture and connatal urinary ascites due to maternal pneumonia and intensive care. The use of blue dye is an effective alternative method to any contrast media radiography and should be considered, especially in very preterm infants.

## 1. Introduction

The presentation of connatal urinary ascites is isolated ascites. The main cause of connatal urinary ascites is intrauterine fetal bladder rupture. Bladder rupture occurs predominantly in fetuses with malformations, of which males with posterior urethral valves make up the majority. Although less often affected, female fetuses preferably suffer from spontaneous bladder rupture, which is a very rare condition [1, 2]. We here report on a very preterm infant suffering from connatal urinary ascites after intrauterine bladder rupture. We are the first to show that using blue dye is an easy-to-perform and noninvasive means of diagnosing a urinary bladder leak.

## 2. Case Presentation

**Figure 1:**
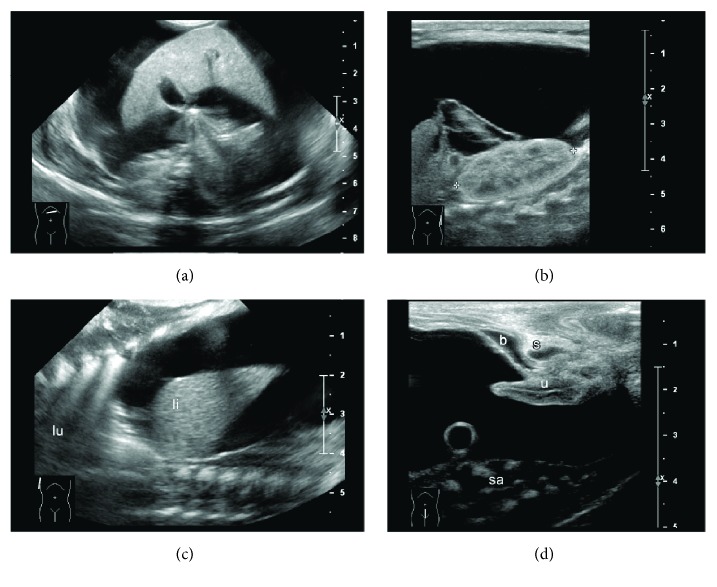
Abdominal ultrasound images showing massive ascites. (a) Transverse section through the epigastrium. (b) Longitudinal section through the left kidney that appears normal in shape and length. (c) Longitudinal section through the liver and lung (li: liver; lu: lung) showing no pleural effusion. (d) Longitudinal section through the bladder (b: bladder; u: uterus; sa: sacrum; s: symphysis), which is almost empty.

**Figure 2:**
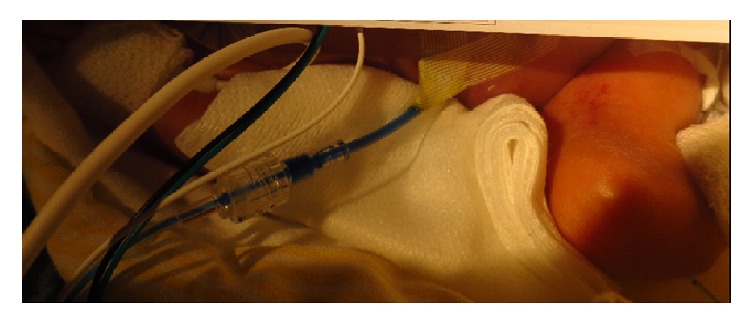
Infant in supine position, ascitic drain in the right abdomen filled with blue dye.

The pregnancy of a 34-year-old, Caucasian fourth gravida was uneventful until 27 + 6 weeks of gestation, when the mother was admitted to a regional hospital with respiratory infection. The woman suffered from H1N1 influenza and experienced respiratory insufficiency. She was transferred to a central hospital intensive care unit. While the mother was ventilated and sedated for ten days, fetal condition was monitored closely by ultrasound examination every other day. At 28 + 1 weeks, the female fetus showed adequate growth (estimated fetal weight: 1116 grams, P50) and Doppler measurements. Of interest, a fetal megacystis was evident (bladder sagittal length: 6.2 cm), but no malformations were evident. At 28 + 2 weeks, the megacystis was less pronounced. In addition, moderate, isolated ascites was present. Two days later the fetal urinary bladder was small, ascites was moderate, and cyst formation was seen adjacent to the bladder and the abdominal wall; in the following week, the cyst was no longer present. Both bladder size and ascites quantity remained constant. At 29 + 4 weeks the amount of the fetal ascites had doubled. Doppler ultrasound and cardiotocography measurements indicated deterioration of fetal condition. The infant was thus delivered via caesarean section. At birth the girl presented with respiratory distress and a severely distended abdomen. APGAR scores were 3/7/8, and umbilical cord arterial pH was 7.27. Birth weight was 1570 grams (P65) and head circumference was 27.5 cm (P50). After initial stabilisation, intubation, and surfactant treatment, high-frequency oscillation was commenced. At bedside, an abdominal ultrasound examination was performed. The ultrasound (Figure 1) showed extensive, isolated ascites, a small urinary bladder normal in appearance, and an unremarkable female genitourinary tract. The subsequent treatment was ascites drainage. Biochemical analysis of the drained clear yellow fluid indicated its urinary nature (creatinine level: 2.17 mg/dl). Because of anuria despite normal blood pressure, an in-out urinary catheter was inserted six hours after birth. The patient remained oliguric and the ascites recurred until a Foley catheter was inserted. As we suspected a connection between the bladder and the peritoneal cavity, we instilled blue dye intravesically. Leakage of dye from the ascitic drain confirmed the connection (Figure 2). With the blocked catheter in place, urine production immediately normalised. Fourteen days later the catheter was removed and spontaneous micturition was unremarkable. A micturating cystourethrogram at age 5 weeks was also without pathological findings. After an uneventful further clinical course, the patient was discharged home at the age of 39 + 3 weeks. Follow-up examinations until the age of 1 year were uneventful.

## 3. Discussion

We here present a female very preterm infant with connatal urinary ascites after spontaneous intrauterine bladder rupture. Delivered at 29 + 4 weeks, our patient is the most preterm female with this complication reported so far [1–3]. The highlight of this case is the use of blue dye to easily and rapidly diagnose a bladder leak.

In females without genitourinary malformations bladder rupture is the most likely cause of urinary ascites [1]. The diagnosis of urinary ascites is confirmed if creatinine is detected in the ascitic fluid [2, 4, 5] and if the ascitic fluid creatinine : plasma creatinine ratio is higher than 1.0 [4, 5]. In our case the ascitic fluid creatinine : plasma creatinine ratio was 1.8 (plasma creatinine level: 1.2 mg/dl), which according to the literature confirms urinary ascites. Our patient's prenatal course was suspicious of bladder rupture. Prenatal findings were a megacystis without malformations, decreasing fetal bladder size accompanied by an increasing amount of fetal ascites, and cyst formation adjacent to the bladder. The infant's postnatal clinical course indicated the persistent patency of a bladder leak.

In previous reports, bladder rupture was demonstrated by ultrasound, micturating cystourethrogram, or computed tomography [1–4]. Due to prematurity, bedside ultrasound was the diagnostic means of choice, but failed to show a bladder wall defect. We then instilled saline into the bladder via the urinary catheter to demonstrate fluid flow from the bladder into the peritoneal cavity using Doppler. This also failed. Finally, with a urinary catheter and an ascitic drain in place, we instilled blue dye into the urinary bladder. Its prompt appearance from the ascitic drain confirmed the connection (Figure 2). This is the first report of the use of blue dye in this context and it offered prompt and accurate diagnosis.

The pathogenesis of spontaneous fetal bladder rupture is unclear. With a prenatal history of maternal H1N1 infection, respiratory failure and intensive care, fetal urinary bladder rupture, and connatal ascites, our case shares similarities with a previous report [2]. We excluded fetal H1N1 infection by negative PCR. Except for this repeated coincidence, there is no evidence for a direct fetal effect of H1N1. Other reported associated factors are certain medications administered to pregnant women. It has been previously discussed that benzodiazepines, opioids [2], corticosteroids, and magnesium sulphate [1] may play a role in the pathogenesis of fetal bladder rupture, because they cross the placenta. Maternal intensive care, in our case, included both benzodiazepines and opioids. One might speculate that the muscle-relaxant effect of benzodiazepines contributed to enlargement of the bladder until a megacystis was present and that opioids induced a spasm of the urinary bladder sphincter leading to urethral obstruction [2]. The proposed mechanism, how corticosteroids and magnesium sulphate administered to mothers prior to premature delivery cause bladder rupture, is similar [1]. In our case, the mother received corticosteroids, but not magnesium sulphate. Whether corticosteroids contributed to fetal bladder rupture remains unclear.

Finally, ischemia of the urinary bladder wall is reported to be an associated factor in the pathogenesis of fetal and neonatal bladder rupture [3–5]. One might speculate that maternal respiratory failure leads to fetal hypoxia and thus to ischemia of the bladder wall. In our case, maternal respiratory compromise may have weakened the fetal bladder wall and predisposed it to rupture. The administration of benzodiazepines and opioids during maternal intensive care possibly caused fetal bladder rupture.

## 4. Conclusion 

In pregnant women requiring intensive care fetuses should be monitored for megacystis and ascites, and fetal bladder rupture should be considered as this may lead to rapid fetal deterioration and preterm birth. Postnatally, accurate diagnosis is important. Our case demonstrates that intravesical administration of blue dye is a noninvasive and fast diagnostic means of confirming a bladder leak. We suggest that the use of blue dye is an effective alternative method to any contrast media radiography and should be considered as first-line diagnostic method in this context.

## Abbreviations

P: Percentile
H1N1: Influenza Serotype H1N1.
